# Preoperative predictors of massive intraoperative bleeding in liver transplant recipients: A retrospective analysis

**DOI:** 10.1097/MD.0000000000045964

**Published:** 2025-11-14

**Authors:** Ran Zhang, Qingqin Tan, Yuanlan Fan, Rui Huang, Quan Wen

**Affiliations:** a Jiangxi Provincial People’s Hospital, The First Affiliated Hospital of Nanchang Medical College, Nanchang, China.

**Keywords:** ALT, AST, blood loss, liver transplantation, massive bleeding

## Abstract

Despite rapid advancements in orthotopic liver transplantation over the past decade, massive intraoperative bleeding (MIB) remains a significant surgical risk for patients. Identifying predictive factors for MIB can optimize preoperative and intraoperative management. We analyzed the predictive value of preoperative laboratory indicators for MIB in liver transplant patients, evaluating their intraoperative blood loss. We collected clinical data from 158 patients who underwent their first liver transplantation between May 1, 2022, and April 30, 2024. Patients were divided into MIB (experimental group: ≥800 mL) and non-MIB (control group: <800 mL) groups based on intraoperative blood loss. We analyzed the correlation between preoperative laboratory indicators and baseline data with massive bleeding. Statistical analyses were performed using the Statistical Package for the Social Sciences software (version 25.0). This study found that the preoperative indices alanine aminotransferase (ALT), aspartate transaminase (AST), total bilirubin, direct bilirubin, albumin (ALB), hemoglobin, hematocrit, prothrombin time, thrombin time, and fibrinogen significantly influenced MIB (*P* < .05). Multivariate logistic regression analysis identified ALT and AST as independent risk factors for intraoperative bleeding (*P* < .05). Receiver operating characteristic curve analysis revealed areas under the curve of 0.619 (95% confidence interval [CI]: 0.528–0.709) for ALT and 0.684 (95% CI: 0.600–0.767) for AST in MIB prediction. In this study, preoperative ALT and AST were identified as risk factors for MIB during liver transplantation, aiding in the development of a predictive model for intraoperative bleeding risk.

## 1. Introduction

Liver transplantation is currently the most effective treatment for acute or chronic liver failure, primary or secondary liver cancer, and end-stage liver disease.^[1]^ Despite rapid advancements in surgical, anesthetic techniques and increased autologous blood salvage in recent years,^[2,3]^ massive intraoperative bleeding (MIB) remains a challenging issue during liver transplantation because of the complex medical conditions and high surgical risks of some patients.^[4,5]^

MIB in liver transplantation can lead to coagulopathy,^[6]^ tissue hypoxia,^[7]^ acute kidney injury,^[8]^ infection,^[9]^ and shock in patients.^[10]^ Massive bleeding and subsequent blood transfusion can lead to some adverse reactions of blood transfusion.^[11]^ For instance, the transfusion of red blood cells (RBCs) can effect on the activity of the graft through immune regulation, increase the risk of alloimmunization and acute/delayed hemolytic transfusion reactions in patients.^[12–14]^ Moreover, such abrupt hemorrhage further strains blood-product supply and intensifies the logistical burden on the transfusion service.

To mitigate operative risk and refine peri-operative management in liver transplant patients with MIB, accurate preoperative prediction of MIB is essential for timely preparation of blood products. We therefore performed a retrospective analysis of all enrolled patients and stratified them according to intraoperative blood loss. Because acute hemorrhage ≥800 mL can precipitate haemodynamic instability and uncompensated hemorrhagic shock, we adopted this volume as the cutoff, defining the cohorts as “MIB” (≥800 mL) and “non-MIB” (<800 mL).^[15,16]^ By examining the preoperative haematological and biochemical parameters of all patients, we sought to identify independent risk factors for MIB and to develop a center-specific predictive model for MIB in liver transplant patients.

## 2. Design and methods

### 2.1. Basic information

A total of 165 adult patients who underwent orthotopic liver transplantation (OLT) at Jiangxi Provincial People’s Hospital from May 1, 2022, to April 30, 2024, were retrospectively selected, and complete information for 158 patients was obtained. All donor livers were sourced from deceased donors. Data, including gender, age, body mass index, basic medical history (hypertension, diabetes, and hepatitis B) and previous history of upper abdominal surgery, were collected from the medical record information system. The intraoperative blood loss of all the enrolled patients was quantified by adding the suction volume, the volume calculated from the weight difference of blood-soaked gauze (assuming 1 g ≈ 1 mL), and the volume in the drainage reservoir.

This study has been approved by the Medical Ethics Committee of Jiangxi Provincial People’s Hospital (the First Affiliated Hospital of Nanchang Medical College) (No. 2025 (24)), and patient informed consent was waived due to the retrospective nature of the analysis.

### 2.2. Inclusion and grouping criteria

The inclusion criteria were as follows: Adult patients aged ≥ 18 years who underwent OLT and had complete clinical data. Exclusion criteria: Pediatric liver transplant recipients, multi-organ combined transplantation recipients, and patients whose medical records were incomplete.

Grouping criteria: All enrolled patients were divided into 2 groups: the experimental group, with intraoperative blood loss ≥ 800 mL, and the control group, with intraoperative blood loss < 800 mL.

### 2.3. Instruments and reagents

A Sysmex automatic blood analyzer with matching reagents, a sysmex automatic blood coagulation analyzer with matching reagents, and an automatic biochemical analyzer with matching reagents were utilized for patient blood and biochemical analyses. The equipment used for cross-matching included a blood group card centrifuge, an incubation chamber, and a microcolumn gel card. Whole blood samples containing heparin anticoagulant were used for the cross-matching test before transfusion.

### 2.4. Data management

Through the medical record and the blood transfusion management systems, we collected the preoperative and postoperative blood and biochemistry-related laboratory data and the transfusion of various blood products during surgery for all patients enrolled over the 2 years. The length of hospital stay, duration of intensive care unit (ICU) admission, and patient outcomes were recorded as short-term prognostic indicators. The baseline data of the 2 groups were compared, and the intraoperative blood transfusion matching mode was observed. Postoperative changes in blood-related indices were also monitored in both groups. Blood-related laboratory indices included hemoglobin (Hb), hematocrit (Hct), platelet count (PLT), activated partial thromboplastin time (APTT), prothrombin time (PT), thrombin time test (TT), and fibrinogen (Fib). Biochemical-related laboratory tests included alanine aminotransferase (ALT), aspartate transaminase (AST), total bilirubin (TBIL), direct bilirubin (DBIL), albumin (ALB), and others. The dose of all blood products transfused was calculated using the international standard unit (U). 1 U of washed RBCs was all prepared from 200 mL of whole blood, 1 U cryoprecipitate or concentrated platelet was prepared by separating fresh frozen plasma from 200 mL of whole blood. Furthermore, 100 mL of plasma was calculated as 1 U. In general, red-cells transfusion is considered when the Hb concentration is ≤7.8 g/dL, the model for end-stage liver disease score is ≥38, the hepatic venous pressure gradient exceeds 16 mm Hg; INR > 1.4, PT exceeds 1.5 times the normal value, plasma transfusion should be considered; Cryoprecipitate is co-administered when the Fib level is <150 mg/dL or when intraoperative blood loss exceeds 1000 mL; Platelet products transfusion is reserved for liver transplant patients whose PLT is <50 × 10^9^/L.

### 2.5. Statistical methods

The Statistical Package for the Social Sciences software (version 25.0) was used for statistical analysis. Continuous variables were presented as means ± standard deviation or medians with interquartile ranges, depending on the data distribution. The Mann–Whitney *U* test or *t*-test was applied where appropriate. Count data are presented as the number of cases (%). And the χ^2^ test or Fisher exact test was used to compare groups. We also took MIB as the dependent variable and used binomial logistic multivariate regression analysis to analyze the independent influencing factors of MIB.

## 3. Results

### 3.1. Preoperative data analysis in liver transplant patients: massive versus non-massive intraoperative bleeding groups

Among the 158 liver transplant patients in the study, the median age was 57 years (interquartile range 43–57) and 138 (87.3 %) were male. The baseline data, blood, coagulation, and biochemical data of the experimental group and the control group revealed that the proportions of males and females, the proportions of patients with underlying diseases were similar between the 2 groups, and that the history of upper abdominal surgery, age, body mass index, PLT, and APTT was not significantly different between the 2 groups. However, compared with the control group, the massive bleeding group presented significant statistical differences in preoperative ALT, AST, TBIL, DBIL, ALB, Hb, Hct, PT, TT, and Fib levels (*P* < .05). Multivariate logistic regression analysis was used to identify the independent risk factors for massive intraoperative blood loss. The results revealed that only ALT and AST were significantly different (Tables 1 and 2).

**Table 1:** Baseline data and preoperative laboratory indicators of the 2 groups of patients.

Parameters	MIB group (67)	Non-MIB group (91)	*P*-value
Gender (male/female, n)	58/9	80/11	.802
Underlying diseases (%)	85.1	87.9	.604
The history of upper abdominal surgery (%)	61.2	54.9	.432
Age	48 (41, 57)	50 (44, 57)	.291
BMI (kg/m^2^)	24.17 (22.22, 26.42)	23.53 (21.78, 26.15)	.418
ALT (U/L)	38 (22, 68)	25 (19, 41)	.021
AST (U/L)	54 (39, 124)	38 (29, 55)	<.001
AST/ALT	1.74 (1.29, 2.09)	1.38 (1.10, 1.80)	.014
TBIL (μmol/L)	105 (24, 347)	34.0 (17.5, 105.5)	.001
DBIL (μmol/L)	79 (7.71, 92.8)	8.9 (4.1, 63.6)	<.001
ALB (g/L)	30.6 (28.3, 34.5)	32.8 (29.4, 37.5)	.022
Hb (g/L)	94 (81, 114)	115 (95, 132)	<.001
Hct (L/L)	0.28 (0.23, 0.33)	0.34 (0.28, 0.4)	<.001
PLT (10^9^)	62 (44, 87)	69 (39, 108)	.889
APTT (s)	36.1 (33.38, 49)	36.4 (32.4, 40.9)	.264
PT (s)	17.1 (13.7, 24.8)	14.6 (12.5, 18.2)	.015
TT (s)	19.8 (17.68, 22.65)	18.1 (16.2, 19.8)	.001
Fib (g/L)	1.53 (1.06, 2.34)	1.99 (1.34, 2.78)	.009

ALB = albumin, ALT = alanine aminotransferase, APTT = activated partial thromboplastin time, AST = aspartate transaminase, BMI = body mass index, DBIL = direct bilirubin, Fib = fibrinogen, Hb = hemoglobin, Hct = hematocrit, MIB = massive intraoperative bleeding, PLT = platelet count, PT = prothrombin time, TBIL = total bilirubin, TT = thrombin time test.

**Table 2:** Logistic regression analysis of preoperative laboratory indicators and MIB during OLT.

Variable	β	Standard error	OR	95% CI		*P*-value
				Lower bound	Upper bound	
ALT	−0.02	0.008	0.98	0.964	0.996	.013
AST	0.026	0.009	1.027	1.009	1.044	.002

ALT = alanine aminotransferase, AST = aspartate transaminase, CI = confidence interval, MIB = massive intraoperative bleeding, OLT = orthotopic liver transplantation, OR = odds ratio.

**Figure 1.**
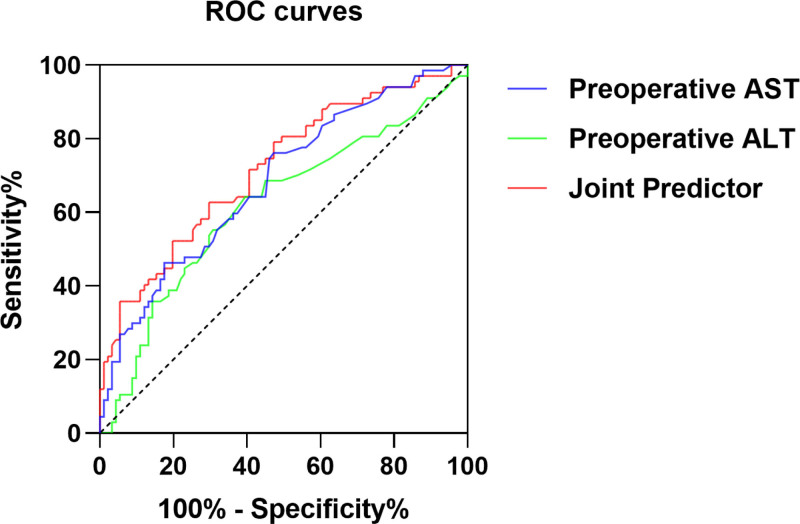
ROC curve analysis of ALT and AST. ALT = alanine aminotransferase, AST = aspartate transaminase, ROC = receiver operating characteristic curve.

We performed receiver operating characteristic (ROC) curve analysis to assess the ability of ALT and AST to predict MIB. The area under the curve (AUC) of ALT was 0.619 (95% confidence interval (CI): 0.528–0.709), and the optimal cutoff value was 28.5 U/L (sensitivity 64.2%, specificity 60.4%). The AUC for AST in predicting MIB was 0.684 (95% CI: 0.600–0.767), which is relatively high. The corresponding cutoff value was 38.5 U/L, with a sensitivity of 76.1% and specificity of 52.7%. The AUC for the combined prediction of ALT and AST was 0.718. These findings suggest that the combined prediction has greater power in predicting MIB (Fig. 1).

### 3.2. Blood transfusion volume and matching ratio of the 2 groups of patients

Compared with the control group, patients in the massive intraoperative hemorrhage group required more RBCs, plasma, and cryoprecipitate during liver transplantation (*P* < .001), and those in the massive intraoperative hemorrhage group exhibited significantly higher transfusion ratios of RBCs to plasma and RBCs to cryoprecipitate (*P* < .01) (Table 3).

**Table 3:** Intraoperative transfusions of various blood products in the 2 groups of patients.

Blood products	MIB group (67)	Non-MIB group (91)	*P*-value
RBCs	8.0 (5.0, 10.0)	4.0 (2.0, 5.5)	<.001
Plasma	10.0 (8.0, 14.0)	6.0 (5.5, 8.0)	<.001
Platelet	0 (0, 0)	0 (0, 0)	.927
Cryoprecipitate	31.00 (20.00, 40.50)	20.00 (20.00, 31.00)	<.001
RBCs/plasma ratio	0.70 (0.57, 1.00)	0.61 (0.33, 0.79)	.004
RBCs/cryoprecipitate ratio	0.24 (0.15, 0.39)	0.15 (0.10, 0.21)	<.001

MIB = massive intraoperative bleeding, RBCs = red blood cells.

### 3.3. Changes in laboratory indices before and after surgery in the 2 groups

Patients in both the experimental and control groups exhibited significant decreases in routine blood indices (Hb, Hct, and PLT) from the preoperative to postoperative periods (*P* < .01). The coagulation-related indicators (APTT) increased after surgery, and only the change of APTT in the control group was statistically significant (*P* < .001). However, the change of PT before and after surgery was significantly different in the MIB group (*P* < .01). The changes of Fib in the 2 groups were significantly increased after the operation (*P* < .05).

Compared with the control group, the changes of postoperative Hb, Hct, PLT, Fib, and APTT in the massive hemorrhage group were significantly different (*P* < .05), the first 4 parameters were significantly lower, and APTT was significantly higher (*P* < .001) (Table 4).

**Table 4:** Comparison of preoperative and postoperative laboratory indicators between the 2 groups.

Time	Hb (g/L)	Hct (L/L)	PLT (*10^9^)	APTT (s)	PT (s)	TT (s)	Fib (g/L)
MIB group							
Preoperative	94.00 (81.00, 114.00)	0.28 (0.23, 0.33)	62.00 (44.00, 87.00)	36.10 (33.38, 49.00)	17.10 (13.70, 24.80)	19.80 (17.68, 22.65)	1.53 (1.06, 2.34)
Postoperative	87.00 (75.00, 95.00)**	0.25 (0.22, 0.28)**	41.00 (30.00, 72.00)***	46.40 (36.30, 61.60)	15.70 (13.50, 17.90)**	18.90 (17.70, 23.30)	1.88 (1.57, 2.34)*
Non-MIB group							
Preoperative	115.00 (95.00, 132.00)	0.34 (0.28, 0.40)	69.00 (39.00, 108.00)	36.40 (32.40, 40.90)	14.60 (12.50, 18.20)	18.10 (16.20, 19.80)	1.98 (1.34, 2.77)
Postoperative	98.00 (85.00, 112.00)***	0.30 (0.25, 0.33)***	54.00 (32.00, 81.00)***	42.20 (36.50, 54.10)***	14.60 (13.50, 16.60)	17.80 (15.90, 21.10)	2.19 (1.85, 2.60)*
*P*-value (MIB vs non-MIB, postoperative)	<.001	<.001	<.001	<.001	>.05	>.05	<.05

Within-group comparisons (postoperative vs preoperative within the MIB or Non-MIB groups) are indicated by asterisks. The absence of symbols indicates no statistically significant difference.

APTT = activated partial thromboplastin time, Fib = fibrinogen, Hb = hemoglobin, Hct = hematocrit, MIB = massive intraoperative bleeding, PLT = platelet count, PT = prothrombin time, TT = thrombin time test.

* *P* < .05.

** *P* < .01.

*** *P* < .001.

### 3.4. Prognostic outcomes of the 2 groups of patients

Compared with the control group, patients in the MIB group had a significantly longer ICU stay (*P* < .001). The MIB group also demonstrated a higher rate of critical illness and mortality, although these differences were statistically non-significant. Additionally, there was no significant difference in the total length of hospital stay between the 2 groups (Table 5).

**Table 5:** Prognostic outcomes of the 2 groups of patients.

	MIB group (67)	Non-MIB group(91)	*P*-value
Length of stay	22.00 (18.00, 29.00)	22.00 (18.00, 28.00)	.529
Length of ICU stay	6.00 (5.00, 9.00)	5.00 (5.00, 5.00)	<.001
Outcome			
Get better (n)	88.05%(59)	93.41%(85)	.242
Critical illness and death (n)	11.94%(8)	6.59%(6)	

ICU = intensive care unit, MIB = massive intraoperative bleeding.

## 4. Discussion

Although numerous studies have explored the predictors of bleeding and transfusion requirements during liver transplantation, Modanlou et al proposed that donor age and recipient serum creatinine could be predictive indicators for intraoperative transfusion in liver transplant recipients.^[17]^ In 2014, Cywinski et al Conducted a retrospective study involving 804 patients with OLT and found that preoperative PLT, model for end-stage liver disease score, creatinine level, and TBIL level were significantly associated with intraoperative transfusion.^[12]^ In summary, studies investigating the predictors of intraoperative transfusion in liver transplant patients have focused primarily on preoperative hematological and coagulation indicators, which are limited. Another important point that has been overlooked is that many may confuse the concepts of massive bleeding and massive transfusion. Massive bleeding is not always supported by massive transfusion. Due to the varying blood bank management specifications, the lack of uniform standards for clinical blood transfusion, and different anesthesia teams following different intraoperative blood transfusion guidelines, the amount of blood products required can vary by 2 to 3 times.^[13]^ China has faced a severe blood shortage of blood in the last 2 years. Therefore, not all cases of MIB during liver transplantation can be compensated with massive transfusions. As a result, it seems unreliable to establish a model for predicting intraoperative massive transfusion based on preoperative variables. Instead, predicting MIB appears to be more meaningful. Our study primarily aimed to identify the predictors of MIB in OLTs to prevent massive bleeding in advance or take measures such as intraoperative autologous blood salvage.^[14,18]^ Only then do we consider transfusion as a remedial measure.

In this study, we grouped patients who underwent OLT according to their degree of intraoperative blood loss. Patients with blood loss ≥ 800 mL were assigned to the massive bleeding group, whereas those with <800 mL blood loss were assigned to the non-massive bleeding group. This study analyzed preoperative factors (laboratory indicators measured 24 hours before surgery) that influence MIB in OLTs. The results revealed that ALT, AST, TBIL, DBIL, ALB, Hb, Hct, PT, TT, and Fib indicators were all associated with MIB. This finding was consistent with the findings of Yuasa et al,^[19]^ who proposed that TBIL and DBIL were risk factors for massive blood loss during liver transplantation. Meanwhile, preoperative PT was considered to be significant for predicting blood transfusion during liver transplantation.^[20]^ However, there has long been controversy over the predictive value of preoperative coagulation test indicators for intraoperative bleeding in transplantation.^[21–23]^ Subsequently, we conducted a binary logistic regression analysis to identify the independent risk factors associated with MIB. The results revealed that only the preoperative indicators ALT and AST were statistically significantly different (*P* < .05). And the combined predictive value of ALT and AST is greater. Statistics on blood usage during surgery for all patients revealed that most patients received a combination of 2 or more blood products, and the amount of blood products used in the massive bleeding group was significantly greater than that in the control group. However, intraoperative platelet transfusion was less, as platelet transfusion during liver transplantation was considered a significant risk factor for postoperative survival.^[24]^ Previous studies have indicated that MIB has an adverse affect on patient outcomes.^[19]^ We also compared the length of hospital stay, the duration of ICU admission, and the outcomes between the massive and the non-MIB groups. The results revealed that the duration of ICU admission was significantly longer in the MIB group than in the control group (*P* < .001), and the incidence of critical illness and mortality was higher in the experimental group than in the control group, although the difference was statistically non-significant.

Despite including various preoperative hematologic and biochemical variables and using rational statistical methods in our study, the sample size was relatively small, and there were center-specific characteristics in blood management and clinical techniques. Therefore, our finding that preoperative ALT and AST were independent risk factors for MIB in OLT should only be considered as a reference for homogenous populations in other medical centers, rather than being directly applicable.

In conclusion, we analyzed that preoperative Hb, Hct, and many other indicators were related to MIB during liver transplantation. Among these indicators, preoperative ALT and AST were found to be independent but modest risk factors with good predictive power for massive blood loss during liver transplantation. Admittedly, the model’s modest ROC AUC values underscore the need for greater discriminatory power. We are therefore launching a prospective, multicentre expansion of the cohort to increase the sample size, minimize bias, and enhance both the reliability and generalisability of the predictive algorithm. MIB in liver transplantation is a multifactorial phenomenon resulting from the complex interplay of surgical, anesthetic, and patient-specific factors. Future research should focus on developing individualized hemostatic strategies and exploring bioengineering solutions to further increase the safety of liver transplantation.

## Author contributions

**Data curation:** Ran Zhang, Yuanlan Fan.

**Investigation:** Quan Wen.

**Methodology:** Rui Huang.

**Project administration:** Ran Zhang.

**Software:** Quan Wen.

**Supervision:** Qingqin Tan.

**Visualization:** Yuanlan Fan.

**Writing – original draft:** Ran Zhang.

**Writing – review & editing:** Quan Wen.

## References

[1] O’Leary JG Lepe R Davis GL. Indications for liver transplantation. Gastroenterology. 2008;134:1764–76. 18471553 10.1053/j.gastro.2008.02.028

[2] de Boer MT Molenaar IQ Hendriks HG Slooff MJ Porte RJ. Minimizing blood loss in liver transplantation: progress through research and evolution of techniques. Dig Surg. 2005;22:265–75. 16174983 10.1159/000088056

[3] Tischer S Miller JT. Pharmacologic strategies to prevent blood loss and transfusion in orthotopic liver transplantation. Crit Care Nurs Q. 2016;39:267–80. 27254642 10.1097/CNQ.0000000000000120

[4] Cleland S Corredor C Ye JJ Srinivas C McCluskey SA. Massive haemorrhage in liver transplantation: consequences, prediction and management. World J Transplant. 2016;6:291–305. 27358774 10.5500/wjt.v6.i2.291 PMC4919733

[5] Wheatley T Veitch PS. Effect of blood transfusion on postoperative immunocompetence. Br J Anaesth. 1997;78:489–92. 9175959 10.1093/bja/78.5.489

[6] Ledgerwood AM Blaisdell W. Coagulation challenges after severe injury with hemorrhagic shock. J Trauma Acute Care Surg. 2012;72:1714–8. 22695446 10.1097/TA.0b013e318245225c

[7] Gutierrez G Reines HD Wulf-Gutierrez ME. Clinical review: hemorrhagic shock. Crit Care. 2004;8:373–81. 15469601 10.1186/cc2851 PMC1065003

[8] Brodsky SV Satoskar A Chen J. Acute kidney injury during warfarin therapy associated with obstructive tubular red blood cell casts: a report of 9 cases. Am J Kidney Dis. 2009;54:1121–6. 19577348 10.1053/j.ajkd.2009.04.024

[9] Freire MP Soares Oshiro IC Bonazzi PR. Surgical site infections in liver transplant recipients in the model for end-stage liver disease era: an analysis of the epidemiology, risk factors, and outcomes. Liver Transpl. 2013;19:1011–9. 23744748 10.1002/lt.23682

[10] Reynolds BR Forsythe RM Harbrecht BG; Inflammation and Host Response to Injury Investigators. Hypothermia in massive transfusion: have we been paying enough attention to it? J Trauma Acute Care Surg. 2012;73:486–91. 23019675

[11] Boyd SD Stenard F Lee DK Goodnough LT Esquivel CO Fontaine MJ. Alloimmunization to red blood cell antigens affects clinical outcomes in liver transplant patients. Liver Transpl. 2007;13:1654–61. 18044783 10.1002/lt.21241

[12] Cywinski JB Alster JM Miller C Vogt DP Parker BM. Prediction of intraoperative transfusion requirements during orthotopic liver transplantation and the influence on postoperative patient survival. Anesth Analg. 2014;118:428–37. 24445640 10.1213/ANE.0b013e3182a76f19

[13] Hevesi ZG Lopukhin SY Mezrich JD Andrei AC Lee M. Designated liver transplant anesthesia team reduces blood transfusion, need for mechanical ventilation, and duration of intensive care. Liver Transpl. 2009;15:460–5. 19399745 10.1002/lt.21719

[14] Pinto MA Grezzana-Filho TJM Chedid AD. Impact of intraoperative blood salvage and autologous transfusion during liver transplantation for hepatocellular carcinoma. Langenbecks Arch Surg. 2021;406:67–74. 33025077 10.1007/s00423-020-01997-7

[15] Si S Liu L Huang J. Location of hemangioma is an individual risk factor for massive bleeding in laparoscopic hepatectomy. JSLS. 2021;25:e2021.00070. 10.4293/JSLS.2021.00070 PMC8678761 34949907

[16] Pan X Gu C Wang R Zhao H Shi J Chen H. Initial experience of robotic sleeve resection for lung cancer patients. Ann Thorac Surg. 2016;102:1892–7. 27623274 10.1016/j.athoracsur.2016.06.054

[17] Modanlou KA Oliver DA Grossman BJ. Liver donor’s age and recipient’s serum creatinine predict blood component use during liver transplantation. Transfusion. 2009;49:2645–51. 19682344 10.1111/j.1537-2995.2009.02325.x

[18] Lee J Park S Lee JG Choo S Koo BN. Efficacy of intraoperative blood salvage and autotransfusion in living-donor liver transplantation: a retrospective cohort study. Korean J Anesthesiol. 2024;77:345–52. 38467466 10.4097/kja.23599 PMC11150109

[19] Yuasa T Niwa N Kimura S. Intraoperative blood loss during living donor liver transplantation: an analysis of 635 recipients at a single center. Transfusion. 2005;45:879–84. 15934985 10.1111/j.1537-2995.2005.04330.x

[20] Araújo T Cordeiro A Proença P Perdigoto R Martins A Barroso E. Predictive variables affecting transfusion requirements in orthotopic liver transplantation. Transplant Proc. 2010;42:1758–9. 20620517 10.1016/j.transproceed.2009.10.007

[21] De Santis GC Brunetta DM Nardo M. Preoperative variables associated with transfusion requirements in orthotopic liver transplantation. Transfus Apher Sci. 2014;50:99–105. 24291115 10.1016/j.transci.2013.10.006

[22] Milan Z Cirkovic A Macmillan J Zaky M Pereira JFHB. Hemostatic markers as predictors of massive blood transfusion in orthotopic liver transplantation. Transplant Proc. 2022;54:734–7. 35249731 10.1016/j.transproceed.2022.01.024

[23] Steib A Freys G Lehmann C Meyer C Mahoudeau G. Intraoperative blood losses and transfusion requirements during adult liver transplantation remain difficult to predict. Can J Anaesth. 2001;48:1075–9. 11744582 10.1007/BF03020372

[24] Pereboom IT de Boer MT Haagsma EB Hendriks HG Lisman T Porte RJ. Platelet transfusion during liver transplantation is associated with increased postoperative mortality due to acute lung injury. Anesth Analg. 2009;108:1083–91. 19299765 10.1213/ane.0b013e3181948a59

